# A Health Equity Problem for Low Income Children: Diet Flexibility Requires Physician Authorization

**DOI:** 10.16966/2380-5528.105

**Published:** 2015-08-20

**Authors:** Jodi D Stookey

**Affiliations:** Children's Hospital of Oakland Research Institute, Oakland, CA, USA

**Keywords:** Child, Nutrition, Obesity

## Abstract

USDA programs, such as the Child and Adult Care Food Program (CACFP), School Breakfast Program (SBP), and/or National School Lunch Program (NSLP), enable child care centers and schools to provide free and reduced price meals, daily, to millions of low income children. Despite intention to equalize opportunity for every child to have a healthy diet, USDA program rules may be contributing to child obesity disparities and health inequity. USDA program rules require child care centers and schools to provide meals that include a specified number of servings of particular types of foods and beverages. The rules are designed for the average, healthy weight child to maintain weight and growth. They are not designed for the underweight child to gain weight, obese child to normalize weight, or pre-diabetic child to avoid incident diabetes. The rules allow for only one meal pattern and volume, as opposed to a flexible spectrum of meal patterns and portion sizes. Parents of children who participate in the CACFP, SBP, and/or NSLP do not have control over the amount or composition of the subsidized meals. Parents of overweight, obese, or diabetic children who participate in the subsidized meal programs can request dietary change, special meals or accommodations to address their child's health status, but child care providers and schools are not required to comply with the request unless a licensed physician signs a “Medical statement to request special meals and/or accommodations”. Although physicians are the only group authorized to change the foods, beverages, and portion sizes served daily to low income children, they are not doing so. Over the past three years, despite an overweight and obesity prevalence of 30% in San Francisco child care centers serving low income children, zero medical statements were filed to request special meals or accommodations to alter daily meals in order to prevent obesity, treat obesity, or prevent postprandial hyperglycemia. Low income children have systematically less dietary flexibility than higher income children, because of reliance on free or reduced-price meals, federal food program policy, and lack of awareness that only physicians have authority to alter the composition of subsidized meals in child care centers and schools. Compared with higher income children, low income children do not have equal opportunity to change their daily dietary intake to balance energy requirements.

## Introduction

In the United States (US), it is a national priority to reduce health disparities related to childhood obesity [1]. The American Medical Association recommends medical intervention for obesity prevention and treatment [2]. Public health agencies, including the CDC and WHO, recommend interventions that address Social Determinants of Health and improve health equity [3]. Social Determinants of Health, including policy, the cost of living, beliefs, clinical services, food supply, and social networks systematically determine opportunity for a healthy diet, and subsume individual behavior change efforts. Interventions are sought to *alter* the obesigenic environment and *equalize opportunity* for every child to have a healthy diet.

USDA food programs, including the Child and Adult Care Food Program (CACFP), National School Breakfast Program (SBP), and National School Lunch Program (NSLP), strive to equalize opportunity for a healthy diet for low income children. CACFP provides free and/or reduced-price meals to over 3 million children in child care centers [4]. The SBP serves over 10 million low income children everyday [5]. The NSLP provides free and/or reduced-price meals to over 21 million children in schools [6].

Although the USDA programs intend to “Ensure That *All* of America's Children Have Access to Safe, Nutritious and Balanced Meals” [7], as gatekeepers to healthy food for low income children they are a feature of unequal food access. Low income children depend on subsidized meal programs, unlike higher income children, who have alternative options for accessing healthy food, independent of these gatekeepers.

This article considers how USDA program policy may be contributing to child obesity disparities in the US, given the reliance of low income children on subsidized meals in child care centers and schools, understanding about the biological causes of obesity, child care and school food environment, clinical practice, and public health initiatives. Summer 2015 is an opportune time for attention to potential USDA policy inequity. The 2015 Dietary Guidelines for Americans are due to be released, federal nutrition policy and school meal reimbursement rules will be revised, and child care centers and schools will re-organize meals offered to children.

## USDA Program Policy

The Child and Adult Care Food Program, School Breakfast Program, and National School Lunch Program provide funding, in the form of reimbursement, to child care providers and schools that serve free and/or reduced-price meals to low income children. Meal reimbursement is only provided, however, if the meals meet USDA program rules, which specify that meals must include a particular number of servings of particular types of foods and beverages [8,9]. “The CACFP reimbursement system does not provide partial credit for meals or snacks that meet most of the requirements; they must meet all requirements specified in the meal patterns (p.50) [8]”.

USDA meal reimbursement rules are based on the Dietary Guidelines for Americans and Dietary Reference Intakes. The rules are explicitly designed for the average, healthy weight child to maintain weight and growth [8]. The rules are not designed for underweight children to gain weight, obese children to normalize weight, or pre-diabetic children to avoid incident diabetes. Although the mandated serving sizes differ by age group to accommodate variation in energy intake requirements by age, within age groups there is no systematic accommodation for variation in energy intake requirement. One meal pattern and volume is offered, as opposed to a flexible spectrum of meal patterns and portion sizes. The reimbursement rules do not provide guidance about what to do if a child is losing or gaining weight while enrolled in child care or during the school year, while eating the specified foods and beverages in specified portions, as part of their total diet.

### Biological Causal Mechanism: Dietary *Change* is a Causal Factor for Weight *Change*

According to the Dietary Guidelines for Americans, “the best way for people to assess whether they are eating the appropriate number of calories is to monitor body weight and *adjust* calorie intake and participation in physical activity based on *changes* in weight over time (p.26) [10]”. In absolute units (kcal/d), ‘excess’ energy intake varies within and between people, depending on many factors, including age, sex, body size, fetal programming, health status, and physical activity.

In the scientific literature, it is widely accepted that obesity reflects excess energy intake relative to energy expenditure, sustained over time, and that weight normalization requires a decrease in energy intake relative to energy expenditure, sustained over time. Altering the macronutrient composition of the diet (e.g. lower fat, lower glycemic intake) may also facilitate weight normalization. *Flexibility to adjust intake, i.e. not any one particular absolute intake, is recommended for weight normalization*.

## Child Care and School Food Environment

Annually, between September and June, children spend as many as 40 hours/week in child care or school. Children consume up to two thirds of their weekly meals and snacks in child care or at school [10]. Child care centers and schools are, thus, in a position to offer *sustained* dietary *change* support to children. In many child care centers and schools, meals and snacks are prepared by food vendors, who have professional capacity to design and *adjust* menus.

Child care centers and schools, and parents of children who participate in CACFP, SBP, and/or NSLP do not have authority to change the composition of the subsidized meals. Parents of overweight, obese, or diabetic children who participate in the subsidized meal programs can request special meals and/or accommodations to address their child's health status, but child care providers and schools are not required to comply with the request unless a licensed physician signs a “Medical statement to request special meals and/or accommodations [11]”. Special meals are reimbursed if physicians “describe the medical condition that requires a special meal or accommodation [11]”. Unlike higher income parents, who can afford to prepare or purchase alternative meals, low income parents do not have legal authority to tailor their child's school meal intake to health status.

## Clinical Practice

Best practice guidelines for clinical obesity prevention and treatment in the US include BMI screening schedules, advice about particular nutrition or activity behaviors, and motivational interviewing [1]. Although physicians might use the brief physician-client encounter to concretely prescribe dietary change by authorizing the Special Meal Accommodation form, authorization of this form is not a systematic part of the best practice guidance. Physicians seem unaware of using the Special Meal Accommodation form to address childhood weight and metabolic syndrome, and unaware that only they, not the parents or the schools, can change a low income child's daily intake of foods, beverages, and portion sizes over the school year. Over the past three years, in San Francisco child care centers that serve low income children, zero medical statements were filed to request special meals and/or accommodations to alter daily meals in order to prevent obesity, treat obesity, or prevent postprandial hyperglycemia [San Francisco Head Start and Child Care Health Program annual screening data, 2011-2014]. Physicians, who are not registered dieticians or school food specialists, may furthermore lack training in what meals should and can feasibly be prescribed. Registered dieticians, despite specialization in meal planning and dietary change, are not authorized to order the special meal accommodation.

The lack of flexibility in free and reduced-price meal requirements for child care centers and schools is clinically relevant. Low income obese adolescents (9-12 y) who are motivated to lose weight, continue to have pizza and milk for lunch, instead of a complex salad and water, and do not normalize weight, because of reliance on free school food; Food that must be purchased and/or prepared by the family or child does not compete with free, ready-to eat school meals (unpublished observations from pilot trials). When free, ready-to eat, complex salads and drinking water, are provided as an alternative to school meals, the same children lose weight [12].

## Public Health Initiatives

As USDA program participation significantly improves the dietary intake of low income children, compared with non-participation, current public health initiatives are focused on foods *outside* of school meals and competitive foods [1,3]. Public health professionals, physicians, nutritionists, food vendors, child care and school staff, and families are not already funded or organized to adjust meals *within* CACFP, SBP, and/or NSLP to prevent or treat obesity or diabetes. Given, however, that these programs may be the main or only source of healthy foods for low income children, additional attention to foods *within* these programs meals might have further impact.

## Summary

Compared with higher income children in the US, low income children do not have equal opportunity to change their daily dietary intake to balance energy requirements. Given reliance on free or reduced-price meals, federal food program policy, and lack of awareness that only physicians have authority to alter the composition of subsidized meals in child care centers and schools, low income children have systematically less dietary flexibility than higher income children. It is unknown if/how revision of meal reimbursement rules, physician best practice guidelines, and/or other policy might increase dietary flexibility for low income children. Tapping untapped potential for physicians to authorize dietary flexibility for low income children may help reduce pediatric health disparities.

## Abbreviations

CACFP: Child and Adult Care Food Program
CDC: Centers for Disease Control
NSLP: National School Lunch Program
SBP: School Breakfast Program
USDA: United States Department of Agriculture
WHO: World Health Organization
